# Arsenic accumulation in lentil (*Lens culinaris*) genotypes and risk associated with the consumption of grains

**DOI:** 10.1038/s41598-019-45855-z

**Published:** 2019-07-01

**Authors:** Mohammad Zahangeer Alam, Md. Anamul Hoque, Golam Jalal Ahammed, Rebecca McGee, Lynne Carpenter-Boggs

**Affiliations:** 1 Department of Environmental Science, Faculty of Agriculture, Bangabandhu Sheikh Mujibur Rahman Agricultural University (BSMRAU), Gazipur, 1706 Bangladesh; 2 Department of Soil Science, Faculty of Agriculture, Bangladesh Agricultural University (BAU), Mymensingh, 2202 Bangladesh; 3 College of Forestry, Henan University of Science and Technology, Luoyang, 471023 P.R. China; 4 Grain Legume Genetics Physiology Research, Agricultural Research Service, United States Department of Agriculture, Johnson Hall, Washington State University (WSU), Pullman, WA 99164-6434 USA; 5 Department of Crop and Soil Sciences, Washington State University (WSU), Pullman, WA 99164-6420 USA; 6 Present Address: Department of Environmental Science, Faculty of Agriculture, Bangabandhu Sheikh Mujibur Rahman Agricultural University (BSMRAU), Gazipur, 1706 Bangladesh

**Keywords:** Plant sciences, Natural hazards

## Abstract

Arsenic (As) is a toxic metalloid. As phyto-toxicity is manifested by its accumulation in different tissue types and subsequent growth inhibition in plants. Despite the vital role of leguminous crops in providing proteins to human diets, a little is known about the As accumulation in lentil. In this study, the rate of As uptake and transport from soil to root, shoot and grain of lentil as well as associated risks with the consumption of As contaminated food were examined. Biomass accumulation of lentil genotypes pardina, red chief and precoz drastically decreased when treated with As at 6 mg kg^−1^ concentration in comparison to 0 and 3 mg kg^−1^ As. Quantification of As concentrations following different treatment periods showed that As accumulation in roots and shoots of 0, 3 and 6 mg kg^−1^ As-treated lentil genotypes was statistically different. Arsenic content in grains of red chief genotype was found significantly lower than pardina and precoz. Moreover, As transport significantly increased in roots and shoots compared to the grains. Due to the high concentrations of As in biomass of lentil genotypes, animal as well as human health risk might be associated with the consumption of the As contaminated legume crops.

## Introduction

Arsenic (As) is a carcinogenic metalloid released into the environment from both natural and anthropogenic sources. The transfer of As in soil-plant systems represents one of the principal pathways for human exposure to As^1^. In particular, As contamination in food crops through irrigation water poses a serious threat to food safety. A recent cohort study showed that daily consumption of 500 g cooked rice containing As content above 200 µg/kg can result in genotoxic effects on human^2^. The scale of this environmental poisoning has been expanding year after year, and is greater than any other hazards. The impact of As poisoning is thought beyond the catastrophe relating to the accidents at Bhopal, India, in 1984, and Chernobyl, Ukraine, in 1986^3^. Although the first As-poisoned (arsenicosis) patient was seen in 1983 in West Bengal, India, it came into consideration quite lately and the cause was confirmed in 1993 in Chapai Nawabganj, Bangladesh^4^. So far, As contamination has affected 59 out of 64 districts of Bangladesh, where As levels have been found above the nationally accepted limit (50 ppb). It is estimated that about 35 to 77 million inhabitants of Bangladesh are at risk through the contamination of As in water and food crops^5^. At global scale, more than 100 million people have been estimated to be chronically exposed to As from drinking water. Obviously, the situation is devastating in Bangladesh, India and Pakistan^6–8^. This toxic element (As) is found naturally in all soils throughout the world^9,10^. It is also released into an environment due to human and natural activities. Ground water, mineral ore, geothermal processes and pesticides are the main source of As^11^. Arsenic was detected in food crops grown in As-contaminated soil and/or irrigated with As-contaminated ground water^12^. Contamination of groundwater by As in the Deltaic region, particularly in the Gangetic alluvium of Bangladesh and part of West Bengal, has become one of the world’s most important natural catastrophes^13^. Since many decades, As contaminated groundwater is being used for drinking as well as irrigation, leading to the contamination of food chain^14^. Accumulating evidence suggests that As in rice, lentil and other food sources could contribute to about 30% of the total As ingestion^15^.

Lentil is one of the most ancient cultivated legume crops^16^. Bangladesh, Canada, China, India, Iran, Nepal, Syria, Turkey, and USA are the major lentil growing countries in the world^17^. The total cultivated area in the world is around 4.6 million hectares producing 4.2 million tons of lentil with an average yield of 1.095ton ha^−1^ ^18^. Lentils provide protein and fiber, as well as many vitamins and minerals, such as iron, zinc, folate, and magnesium. In addition, the phytochemicals, saponins, and tannins found in lentil possess antioxidant and anti-carcinogenic properties, indicating that lentils may have significant anti-cancer effects^19^.

Among all lentil growing regions, many of lentil growing countries are highly contaminated by As. Specifically, west Bengal and Bangladesh combined rank the second largest As contaminated region in the world. About 8% of the area in the United States of America is As affected^11^. Despite this, the As element is not-essential and generally toxic to many food crops including lentil crops. Lentil roots are typically the first tissue to be exposed to As, where the metalloid inhibits root extension and proliferation. This metal translocated to the shoot and grain and it can be rigorously constrained of physiological growth by slowing or arresting growth and biomass accumulation, as well as compromising plant reproductive capacity^20^. Osmotic as well as oxidative stress increases in food crops due to the accumulation of As in biomass. Moreover, lipid peroxidation, electrolyte leakage, H_2_O_2_ accumulation, root oxidizability and the activities of antioxidant enzymes change drastically in response to As stress^21^. Arsenic-stressed plants show reduced growth and pigment content. Particularly, total chlorophyll, catalase and ascorbic acid content drastically reduced in food crops due to the imposition of excess metals (As)^22^.

High concentrations of As interfere with critical metabolic processes, which may lead to the death of lentil plants^23–26^. Arsenic ingestion can lead to serious diseases, including cancers^27–29^. However, this metalloid As is universal in the environment due to geological and human activities. Arsenic in the soil is taken up by plants, accumulated in the edible parts (such as lentil grains) and is further expended by humans and other organisms higher in the food chain^29^. Currently, millions of people, especially in South and South-east Asia, are at risk for exposure to food that is contaminated with As^30^. Excessive consumption of As from food crops leads to As accumulation in tissues and inhibits cellular enzyme activities. Inhalation, ingestion and skin contact are the primary routes of human exposure to the As. Chronic As ingestion is known to cause skin cancer, and there is substantial evidence that it increases risk for cancers of the bladder, lung, kidney, liver, colon, and prostate. Recent studies have also shown that As is associated with a number of non-neoplastic diseases, including cardiac disease, cerebrovascular disease, pulmonary disease, diabetes mellitus and diseases of the arteries, arterioles, and capillaries^31^. Individuals with chronic Hepatitis B infection, protein deficiency or malnutrition may be more sensitive to the effects of As^32^.

Reduction of As concentrations in soils and food crops is significantly important for ensuring sustainable crop production as well as food safety. In this regard, phytoremediation of As with lentil crops depends on in-depth understanding of As transport in lentil plants. In the present study, we hypothesized that As uptake, transport and accumulation might differ among different genotypes of lentils such as precoz, red chief and pardina, which might also show differential impacts on nutritional quality and associated health risk with the consumption of As- contaminated grains. In this assessment, we conducted a research on As uptake in root, shoot and grains of lentil crops and the association of risk with the consumption of As-contaminated lentil.

## Results

### Dry weight of root, shoot and pod

**Figure 1:**
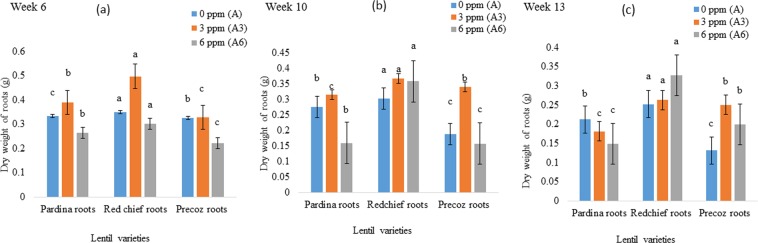
Effect of As uptake on the dry weight (Mean ± SEM) of lentil roots at (**a**) week 6; (**b**) week 10; and (**c**) week 13. Arsenic treatments indicate concentration of As in peat moss; A, control, 0 mg As kg^−1^ peat moss; A_3_, 3.0 mg As kg^−1^ peat moss; A_6_, 6.0 mg As kg^−1^ peat moss. Means denoted by different letters under the same As level indicate significant difference at 0.1% level of significance.

**Figure 2:**
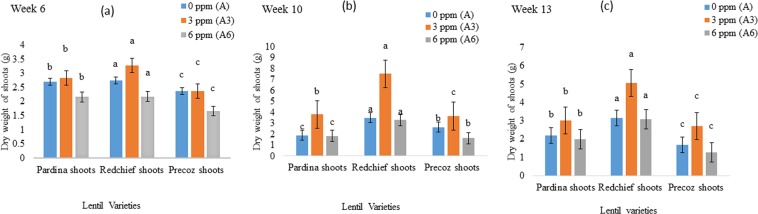
Effect of As uptake on the dry weight (Mean ± SEM) of lentil shoots at (**a**) week 6; (**b**) week 10; and (**c**) week 13. Arsenic treatments indicate concentration of As in peat moss; A, control, 0 mg As kg^−1^ peat moss; A_3_, 3.0 mg As kg^−1^ peat moss; A_6_, 6.0 mg As kg^−1^ peat moss. Means denoted by different letters under the same As level indicate significant difference at 0.1% level of significance.

**Figure 3:**
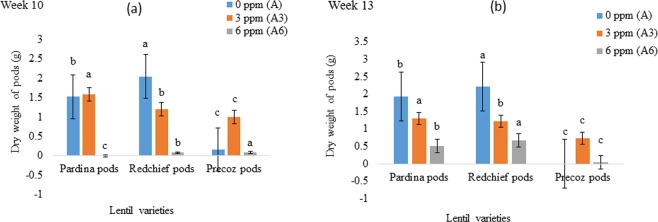
Effect of As uptake on the dry weight (Mean ± SEM) of lentil pods at (**a**) week 10; (**b**) week 13. Arsenic treatments indicate concentration of As in peat moss; A, control, 0 mg As kg^−1^ peat moss; A_3_, 3.0 mg As kg^−1^ peat moss; A_6_, 6.0 mg As kg^−1^ peat moss. Means denoted by different letters under the same As level indicate significant difference at 0.1% level of significance.

Dry weights of pardina roots were found 0.333, 0.389 and 0.264 g in 0, 3 and 6 mg kg^−1^ As treated pots, respectively at week 6. Similarly, dry weights of red chief roots were found 0.349, 0.497 and 0.301 g at 0, 3 and 6 mg kg^−1^ As treated pots, respectively. Chronologically, average dry weights of root, shoot and pod were found lower in 6 mg kg^−1^ As treated lentil cultivars at week 6, 10 and 13. On the other hand, average dry weight of red chief root, shoot and pod at 0, 3 and 6 mg kg^−1^ As treated condition were found significantly higher than other lentil genotypes at week 6, 10 and 13 (Figs 1a–3b). Treatment and lentil varieties both showed significant differences on the dry weight of root at week 6. Similarly, treatments and varieties both were found significantly different on the effect of dry weight of lentil shoot at week 6, 10 and 13. In week 10, treatment and varietal effects on the dry weight of pod were found statistically different. On the other hand, only treatment effect was found significantly different on the pod dry weight at week 13 (Table 1).

**Table 1:** Significance test on the dry weight of lentil root, shoot and pod at different As treated lentil plant at week, 6, 10 and 13.

Week	Root			Shoot		Pod	
	Factors	F value	Pr(>F)	F value	Pr(>F)	F vale	Pr(>F)
Week 6	Treatment	3.80	0.0418*	4.32	0.0292*	—	—
	Variety	9.34	0.0016**	5.028	0.018*	—	—
	Treatment: Variety	0.86	0.502	0.504	0.73	—	—
Week 10	Treatment	1.67	0.215	12.35	0.00042***	2.73	0.092^@^
	Variety	1.62	0.223	16.85	7.509e-05***	8.87	0.0020**
	Treatment: Variety	0.49	0.741	1.85	0.162	1.87	0.1580
Week 13	Treatment	2.07	0.154	5.148	0.017*	3.43	0.054^@^
	Variety	0.209	0.813	3.49	0.052^@^	2.33	0.125
	Treatment: Variety	0.61	0.657	0.178	0.94	0.91	0.476

***Indicates significant difference at *p* < *0*.*001* level of significance **Indicates significant difference at *p* < *0*.*01* level of significance, *Indicates significant difference at *p* < *0*.05 level of significance, (@) Indicates significant difference at *p* < *0*.*1* level of significance.

### Arsenic accumulation in root

Treatment, interaction result of variety and treatment on As uptake in root of lentil genotypes were found significantly different at week 6 and 10. On the other hand, only treatment effect on As accumulation in root was found significantly different at week 13 (Table 2). Arsenic accumulation in root was found significantly higher at A_6_ (6 mg kg^−1^) treated pardina, red chief and precoz lentil genotypes than control (0 mg kg^−1^) and A_3_ (3 mg kg^−1^) during week 6, 10 and 13. Based on the treatment period, accumulation of As in root of control, A_3_ and A_6_ treated lentil genotypes were found statistically different. For instance, As uptake in root of pardina, red chief and precoz lentil genotypes at week 6 was found significantly higher than week 10 and 13 (Table 3). However, As uptake was found at similar rate during week 6 and 10 in root of these lentil genotypes. In week 13, As uptake in red chief genotype was lower than precoz and pardina (Table 3). Arsenic uptake in roots in A_6_ treated lentil genotypes was more than other As treatments.

**Table 2:** Significance test for the accumulation of As in root, shoot and grain of lentil genotypes according to the variety and treatment at week 6, 10 and 13.

Week	Root			Shoot		Grain	
	Factors	F value	Pr(>F)	F value	Pr(>F)	F vale	Pr(>F)
Week 6	Treatment	204.5	4.20e-13***	72.705	2.387e-09***	—	—
	Variety	23.0	1.10e-05***	2.716	0.092^@^	—	—
	Treatment: Variety	22.1	9.6e-07***	1.444	0.260	—	—
Week 10	Treatment	664.2	<2.2e-16***	312.456	1.057e-14***	3.3	0.05^@^
	Variety	56.0	1.859e-08***	1.223	0.317	3.7	0.04*
	Treatment: Variety	21.2	1.281e-06***	0.840	0.517	2.1	0.114
Week 13	Treatment	246.0	8.496e-14***	307.889	1.202e-14***	4.8	0.02*
	Variety	1.6	0.219	1.133	0.344	5.2	0.015*
	Treatment: Variety	0.4	0.753	1.133	0.372	1.6	0.21

***Indicates significant difference at *p* < *0*.*001* level of significance, *Indicates significant difference at *0*.*01* ≤ *p* < *0*.*05* level of significance, (@) Indicates significant difference at *0*.*05* ≤ *p* < *0*.*1* level of significance.

**Table 3:** Arsenic accumulation (mg kg^−1^) in root, shoot and grain in Pardina, Red Chief and Precoz lentil varieties after 6, 10 and 13 weeks of growth.

Variety	Arsenic treatments		Week 6		Week 10			Week 13	
		Root	Shoot	Root	Shoot	Grains	Root	Shoot	Grains
Pardina	Control	0.00 c	0.00 c	0.000 c	0.000 c	0.000	0.000 c	0.000 c	0.000
	A3	1.12 b	0.55 b	0.827 b	0.440 b	0.013	1.298 b	0.522 b	0.009
	A6	3.16 a	1.19 a	1.781 a	1.380 a	0.003	2.781 a	1.380 a	0.005
Red chief	Control	0.000 c	0.000 b	0.000 c	0.000	0.000 b	0.000 c	0.000 c	0.000 b
	A3	1.036 b	0.339 b	0.877 b	0.484	0.0007b	1.155b	0.488 b	0.005 a
	A6	1.447 a	0.841 a	2.548 a	1.267	0.005 a	2.781 a	1.387a	0.006 a
Precoz	Control	0.000 b	0.000 b	0.000 c	0.000 c	0.000	0.000 c	0.000 c	0.000
	A3	1.16 a	0.63 a	1.87 b	0.61 b	0.0007	1.49 b	0.72 b	0.023
	A6	1.41 a	0.92 a	2.78 a	1.38 a	0.004	3.11 a	1.38 a	0.032
Treatments	Control	0.000 a	0.000a	0.000 a	0.000 a	0.000 a	0.000 a	0.000 a	0.000 a
	A3	1.104 b	0.509 b	1.192 b	0.513 b	0.0092 ab	1.316 b	0.577 b	0.012 b
	A6	2.008 c	0.986 c	2.369 c	1.342 c	0.0136 b	2.892 c	1.380 c	0.014 b
Varieties	Pardina	0.868 a	0.579 a	1.359 b	0.606 a	0.005 a	1.426 a	0.634 a	0.0048 a
	Red chief	0.827 a	0.393 a	1.141 b	0.583 a	0.0017 a	1.312 c	0.623 b	0.0038 b
	Precoz	0.858 a	0.522 a	1.537 b	0.666 ab	0.0158 a	1.552 b	0.700 b	0.0185 a

Arsenic treatments indicate concentration of As in peat moss; control, 0 added arsenic; A_3_, 3.0 mg As kg^−1^ peat moss; A_6_, 6.0 mg As kg^−1^ peat moss. Means within each variety and time followed by a different letter indicate significant difference at *p* ≤ *0*.*001*.

### Arsenic accumulation in shoot

Treatment effect on As accumulation in shoot of lentil genotypes was found statistically significant during week 6, 10 and 13 according to significance test. On the other hand, varieties were found statistically different for As uptake in shoot at week 6 rather than week 10 and 13 (Table 2). Arsenic accumulation in the shoot was found higher at A_6_ treated pardina, precoz and red chief lentil genotypes during week 6, 10 and 13 (Table 3). On the other hand, accumulation of As at week 6 and 13 in the shoot of the pardina, and red chief genotype was found significantly different between A_6_ and control, and A_6_ and A_3_ treated lentil genotypes. According to week, accumulation of As in shoot of control, A_3_ and A_6_ treated lentil genotype was found statistically different. Red chief was found significantly different at week 13 for uptake of As in its shoot from week 6 and 10. Arsenic accumulation in shoots of pardina, red chief and precoz genotypes were found statistically insignificant at week 6 and 10 (Table 3). Arsenic uptake in shoot with A_6_ treated lentil genotypes was more than other As treatments (Table 3).

### Arsenic accumulation in grain

Treatment and varietal effects on As accumulation in grains of lentil genotypes were found statistically significant during week 10 and 13 (Table 2). Arsenic accumulation in grains was found statistically insignificant between control, A_3_ and A_6_ treated pardina and precoz lentil genotype during week 10 and 13. At week 10 and 13, As accumulation in grains of red chief genotype was found statistically different between the control and A_6_ treatment. According to week, accumulation of As in grain between control, and A_6_ treated lentil genotypes was found statistically dissimilar. This uptake in the grains was found statistically insignificant between A_3_ and A_6_ treatment. Arsenic uptake in grains of red chief was found significantly lower than pardina and precoz at week 13. Arsenic uptake in grains showed insignificant difference between pardina and precoz (Table 3). Arsenic accumulation in grains was found lower than root and shoot during week 10 and 13. Arsenic in grains increased by 20% and 40% in red chief and precoz with 6 mg As kg^−1^ peat moss as compared to 3 mg As kg^−1^ peat moss after 13 week of growth. Arsenic in grain was found 17% higher by the treatment of 6 mg As kg^−1^ peat moss in comparison to 3 mg As kg^−1^ peat moss after week 13. Red chief genotype was found low As accumulator in contrast to pardina and precoz (Table 3).

## Discussion

Arsenic (As) is a lethal metalloid. Its accumulation in plant tissues and associated health risk with the consumption of As contaminated grains are matters of huge public concern. Among food crops, rice as well as lentil is also grown in As contaminated areas in Bangladesh and other contaminated areas in the world. Arsenic is translocated from soil to root shoot and grains of lentil and other food crops^33^. As a result, As reduces the biomass accumulation of lentil genotypes. Lentil seedlings showed normal growth in As free pots. The seedling growth was negatively affected by increasing the rate of As concentration in pardina, red chief and precoz genotypes. Similarly^34^, conducted research on As toxicity in food crops. They found a high concentration of As decreased the plant growth and development by inducing phyto-toxicity. Due to the As toxicity, germination, plant height, number of roots and shoot growth were negatively affected which might eventually lead to the death of food crops^35–39^. The transportation and accumulation of As in plants followed the order, roots > shoots > grains^40,41^. Arsenic contamination in growing media (0.2 mg kg^−1^) causes negative effects on food crops^42,43^. Similarly, 0.6 mg kg^−1^ As in soil affected soybean growth^44^. Likewise, biomass of 3 mg kg^−1^ and 6 mg kg^−1^ As-treated pardina, red chief and precoz lentil genotypes significantly decreased compared to the control plants grown in As free medium (Figs 1a–3b).

Arsenic is one of the most toxic elements for the reduction of biomass production in food crops. In this experiment, dry weight of lentil genotypes was found lower in As treated lentil crops than As free crops (Figs 1a–3b). It is in agreement with the previous studies as As contamination could reduce dry weight of root and shoot in maize and sunflower plants^45^. Similarly, tomato (*Lycopersicum esculentum*) plants grown under different levels of As show As toxicity. Arsenic exposure resulted in a drastic decrease in plant growth parameters (e.g., maximum decrease of 76.8% in leaf fresh weight) and fruit yield in tomato crops (maximum reduction of 79.6%)^46^. Upon translocation of As can rigorously constrain plant growth by slowing or arresting expansion and biomass production as well as compromising plant reproductive capacity through losses in fertility, yield, and fruit production^20^.

Consequently, As accumulation significantly increased in the root of lentil genotypes from soil solutions. Pardina, red chief and precoz, varieties were shown to have significant uptake of As in their roots (Table 3). The results are in agreement with a study on chickpea (*Cicer arietinum L*.), a major supplementary food in many areas throughout the world^47^. On the other hand, in mangrove plants such as, (*Aegiceras corniculatum* L.), seedlings grown in As contaminated soils, showed increased As concentrations in roots stems and leaves with increasing treatment concentrations of As, but the As accumulation rates in the roots were found 74.54–89.26% of the total As accumulation in the plants^48^. A similar range of As in the roots (14.5–27.4 mg kg^−1^) was found in maize (*Zea mays)*^49^.

Notably, arsenic can be translocated from the roots to the shoots of plants. In the present study, As concentration significantly increased in the shoot of lentil genotypes (Tables 2 and 3). Similarly, As is transported significantly to the shoot, which impairs the growth, and biomass accumulation in rice plants^26,50^. In general, As accumulation in the plant increases with the increasing As concentrations in soils. Nonetheless, terrestrial plants such as legume crops show a higher concentration of As in shoot to root compared with emergent plants^51^.

Arsenic can be accumulated in grains of lentil crops, but this is not a level that is significant for all genotypes (Table 3). In this context, precoz genotype uptakes a significant level of As compared to pardina and red chief cultivars (Table 3). As well as lentil genotypes, As can be accumulated in the edible grains of *Phaseolus vulgaris*. It is also found that increasing the concentration of As in plants decreases the plant growth as well in the lentil plants^52,53^. Research was also conducted on As uptake in several pulse crops such as pea which showed the highest As uptake (1.30 mg kg^−1^)^54^. Arsenic in selected paddy soils of China causes toxicity to bean, lentil, oats and other food crops^55,56^.

Extreme uptake of this metalloid may cause physiological changes in food crops, producing a wide range of detrimental effects, such as suppression of photosynthesis and pigment synthesis, oxidative stress, and other metabolic disturbances^57^. This metal (As) induces oxidative damage in lentil and other food crops due to its excessive uptake in their biomass. Oxidative stress is associated with the increasing levels of reactive oxygen species (ROS) and osmolytes in As stress condition^58^. Although the accumulation of As in roots, shoots were found higher than grains in lentil (Table 3), this element can be transferred to human and animal bodies through the food chains^59^. In human beings, inorganic As species arsenate (AsV) and arsenite (AsIII) are strongly cytotoxic and may lead to As-induced skin hyperkeratosis and cancer^60,61^. In Southern Asia, groundwater contaminated with As is used for the irrigation in food crops^8,62^. Lentil grown on As contaminated soil contains considerable amounts of As in shoots tissues and grains^63^. Persistence of this As within soil and its toxicity to plants, animals and human are of grave concern. Long-term exposure to low concentrations of As can lead to skin, bladder, lung, and prostate cancer. Non-cancer effects of ingesting As at low levels include cardiovascular diseases, diabetes, and anemia^64,65^.

It is well understood that As is a threat for the development of lentil crops in As contaminated regions in the world. For this reason, good agricultural practices are significantly important to get an optimum yield in this food crop^66^. The optimum water and N doses both are important components as recognized good agricultural practices for attaining higher yield, which may enhance grain yield under abiotic stress^66^. As well, optimum temperature and radiation both are important indicator for the increasing of biomass^67^. Good agricultural practices such as optimum temperature, water use efficiency, radiation, and nutrient availability are all affected due to As stress in soils. On the other hand, drought, salinity, lead (Pb), Cd, Cu, Cr and As stress in food crops disrupt the photosynthesis and its associated metabolic activities^57^. This type of stress severely decreases the photosynthesis, water use efficiency, stomatal conductance, chlorophyll contents, and antioxidant defense mechanism in crops^68,69^. Likewise, oxidative damage and osmotic stress increase in food crops due to As toxicity^70,71^. In this situation, mycorrhyzal association might mitigate different abiotic stresses and minimize metal toxicity as well as associated health hazards^72–75^. Mycorrhizal fungi colonized with the root cortex and extended the network of its hyphae into the surrounding environment. These external hyphae can contribute to improving plant nutrients for increasing the biomass growth as well as can alleviate heavy metal toxicity by modulating the metal acquisition in plants from contaminated soils^76^. In addition, agronomic and civil engineering methods such as, judicious use of water, management of soil and plant-nutrients might be recommended in As prone crop growing areas to mitigate the building up of As in human food chain and thus minimizing the negative impact on the environment^77^. Chitosan (CH) and biochar (BC) can be used to reduce mobility and bioavailability of heavy metals and to facilitate plant growth by improving the antioxidant system^78,79^. It is to be noted that biomass of lentil should be toxin free to the end users. If biomass becomes affected in the As contaminated region, the associated risk with the consumption of As contaminated food would definitely increase. In this circumstance, reduction of As transportation from soil to food crops towards the human food chain is significantly important for a sustainable global environment.

## Conclusion

Lentil is an important leguminous crop that provides protein to human diets. Arsenic accumulated in tissues of lentil genotypes and its reallocated to grains enhance health risk with the consumption of contaminated tissue. In this study, we found that concentration of As transport significantly increased from soils to root and shoot tissues and grains in lentil genotypes. Due to such high As transport, biomass of the crops was negatively affected in their entire life cycle. As a result, root and shoot mass of lentil genotypes was found significantly affected. Pardina, red chief and precoz lentil genotypes responded remarkably in terms of As uptake from soils to their root, shoot and grains. Arsenic in grains was increased 17% by the treatment of 6 mg As kg^−1^ peat moss in comparison to 3 mg As kg^−1^ peat moss after 13 week of growth. Thus, the toxic metalloid (As) might transfer to the human body through the consumption of grains, thereby increasing health risks. Therefore, development of As mitigation technologies that could improve plant growth by restricting As transport to plant tissue is urgently needed to expand lentil production in the As contaminated regions throughout the world as well as the reduction of health risk with the consumption of this food crop.

## Methods

### Peat Moss and Pot

Peat moss was collected from the local market in the USA. This growing media was brought to the greenhouse in the Department of Crop and Soil Sciences at Washington State University (WSU) for the growing of different lentil genotypes. The sizes of pots were 275–300 ml volume. All pots were made of toxin free plastic.

### Lentil genotypes and nutrient added from fertilizers

Precoz, red chief, and pardina lentil genotypes were collected from ICARDA (International Center for Agricultural Research in the Dry Areas) for this pot experiment. Slow released mixed fertilizer (Osmocote *plus*) was purchased from the local market in the USA. The ratios of N P K 15: 9: 12 were found in this slow released fertilizer. Out of the 15% total Nitrogen (N), 8.4 and 6.6% were added to its fertilizer from the source of ammoniacal and nitrate nitrogen, respectively. On the other hand, available phosphate (P_2_O_5_), soluble potash (K_2_O), total magnesium (Mg), sulfur (S), boron (B), cupper (Cu), total iron (Fe), manganese (Mn), molybdenum (Mo), and zinc (Zn) at 9, 12, 1.3, 5.9, 0.02, 0.05, 0.46, 0.06, 0.02 and 0.05% were added from this slow released fertilizer in this pot experiment, respectively.

### Sowing of lentil seeds

Precoz, red chief, and pardina seeds were sown in the pots on 13^th^ March, 2014. Seven to 8 seeds from each variety were spread at 2–3 cm depth in peat moss in each pot. After the emergence of the lentil seeds, only 5 seedlings were kept in each pot for further sampling during different stages of lentil plant. Recommended doses (2 g pot^−1^) of slow released fertilizers were applied during seedling and flowering stages of lentil plants.

### Preparation and application of arsenic diluted solution in lentil plants

Sodium arsenate dibasic heptahydrate (Na_2_HAsO_4_.7H_2_O) was used as the source of As. Deionized water was used for the preparation of As diluted solutions. Arsenic solutions were applied from seedling to mature stages at intervals of about every 7 days. About 0.012494 g sodium arsenate dibasic heptahydrate was added to 1liter water for the preparation of 3 mg L^−1^ concentrated As solution. On the other hand, 0.02498 g of sodium arsenate dibasic heptahydrate was taken for the preparation of 6 mg L^−1^ concentrate As dilute solution. These diluted solutions were kept in bottles with proper labeling and preserved in a refrigerator for further application in the pot experiment.

### Treatments and replications in pot experiment

Arsenic free peat moss was used as the growing media in this pot experiment. Three treatments were followed such as, control (A) = 0 mg As kg^−1^ peat moss, A_3_ = 3 mg As kg^−1^ peat moss and A_6_ = 6 mg As kg^−1^ peat moss. Three varieties of lentils, precoz, red chief and pardina were selected for this pot experiment. Three replications with three lentil varieties were followed at week 6, 10 and 13 sampling point and the total number of pots was 81 for this experiment.

### Collection of plant sample at week 6, 10 and 13

Precoz, red chief and pardina seedlings were collected from each of the treatment at week 6, 10 and 13. Three pots including seedlings of each genotypes were removed from each treated tray randomly at week 6. After collection of seedlings, roots were washed with distilled water. Then water was removed from the washed roots using tissue paper. Roots were separated from the shoot of each collected lentil seedlings using scissors. All root and shoot samples were kept separately inside an envelope with proper labeling on each sample. After that, all root and shoot samples were kept in a drying oven for 72 hours at 55–65 °C. Similarly, root, shoot and pod samples of lentil genotypes from each of the treated pots were collected at week 10 and 13 also.

### Dry weight, grinding and sieving of plant samples

Dried samples were brought into the Laboratory of Crop and Soil Sciences at WSU. Dry weight of root, shoot and pod were taken separately. Meanwhile, grains were separated from pod of lentil manually by hand with gloves. Hand gloves were changed during the separation of grain for each sample. Then the samples were ground separately by a coffee grinder using liquid nitrogen. The grinder was cleaned between the samples with ethyl alcohol (C_2_H_5_OH) and tissue papers. These ground root, shoot and grain samples were sieved with 250 µ mesh. Then all samples were kept in envelopes with proper labeling.

### Digestion of samples

Lentil roots, shoots and grains (seeds) were digested separately following heating block digestion procedure^80^. Of the plant samples, 0.1 g ground root, shoot and grains samples were put into clean digestion vessels and 5 ml concentrate HNO_3_ was added to it. The mixture was allowed to stand overnight under a fume hood. On the following day, this vessel was digested in a digestion block for 1 hour at 120 °C. This content cooled and 3 ml HClO_4_ was added to it. Again, samples were put into the heating block for 3–4 hours at 140 °C. Generally, heating stopped whenever white dense fume of HClO_4_ was emitted into air. Then cooled samples were diluted to 25 ml with de-ionized water and filtered using Whatman 42 filter paper. Finally, samples were stored in polyethylene bottles. Prior to samples digestion, all glassware was washed with 2% HNO_3_ followed by rinsing with de-ionized water and drying.

### Analysis of total arsenic

Digested samples were analyzed for the determination of total arsenic in the lentil root, shoot, and grains. The total arsenic in root and shoot tissue and reallocation to grains of the lentil plants was analyzed by flow injection hydride generation atomic absorption spectrophotometry (FI-HG-AAS, Perkin Elmer A Analyst 400-USA) using external calibration^81^. The optimum HCl concentration was 10% v/v and 0.4% NaBH_4_ which produced the maximum sensitivity. For each sample of the digested lentil’s root, shoot, and grains, three replicates were taken and the mean values obtained based on the calculation of those three replicates. Standard Reference Materials (SRM) from the National Institute of Standards and Technology (NIST), USA was analyzed using the same procedures from the start of the experiment, during and at the end of the measurements to ensure continued consistency and accuracy. Method detection limit (MDL) for As was 0.02 µg/l or ppb (parts per billion).

### Statistical analysis

The experiment was carried out following Completely Randomized Design (CRD). Level of significance was analyzed between average dry weight of root, shoot and pod at different As levels in lentil genotypes using software R. Accordingly, the significance test for the comparison of As uptake in root, shoot and grains of lentil plants was performed using software R as well.

## Data Availability

Data supporting the findings of the current study are available from the corresponding author on reasonable request. All data analyzed during this study are included in this published article.
