# Author Correction: Arsenic accumulation in lentil (*Lens culinaris*) genotypes and risk associated with the consumption of grains

**DOI:** 10.1038/s41598-019-54736-4

**Published:** 2019-11-26

**Authors:** Mohammad Zahangeer Alam, Md. Anamul Hoque, Golam Jalal Ahammed, Rebecca McGee, Lynne Carpenter-Boggs

**Affiliations:** 1 Department of Environmental Science, Faculty of Agriculture, Bangabandhu Sheikh Mujibur Rahman Agricultural University (BSMRAU), Gazipur, 1706 Bangladesh; 2 Department of Soil Science, Faculty of Agriculture, Bangladesh Agricultural University (BAU), Mymensingh, 2202 Bangladesh; 3 College of Forestry, Henan University of Science and Technology, Luoyang, 471023 P.R. China; 4 Grain Legume Genetics Physiology Research, Agricultural Research Service, United States Department of Agriculture, Johnson Hall, Washington State University (WSU), Pullman, WA 99164-6434 USA; 5 Department of Crop and Soil Sciences, Washington State University (WSU), Pullman, WA 99164-6420 USA; 6 Present Address: Department of Environmental Science, Faculty of Agriculture, Bangabandhu Sheikh Mujibur Rahman Agricultural University (BSMRAU), Gazipur, 1706 Bangladesh

Correction to: *Scientific Reports* 10.1038/s41598-019-45855-z, published online 01 July 2019

This Article contains errors in Tables 1 and 2, where the columns entitled “F vale” should read “F value”.

